# The impact of common genetic variants in the mitochondrial glycine cleavage system on relevant metabolites

**DOI:** 10.1016/j.ymgmr.2018.05.006

**Published:** 2018-06-11

**Authors:** Jessica O'Reilly, Faith Pangilinan, Karsten Hokamp, Per M. Ueland, John T. Brosnan, Margaret E. Brosnan, Lawrence C. Brody, Anne M. Molloy

**Affiliations:** a Department of Clinical Medicine, School of Medicine, Trinity College Dublin 2, Ireland; b Genetic and Environmental Interaction Section, National Human Genome Research Institute, Bethesda, MD 20892, United States; c School of Genetics and Microbiology, Trinity College, Dublin 2, Ireland; d Section of Pharmacology, Institute of Medicine, University of Bergen and Haukeland University Hospital, 5021 Bergen, Norway; e Department of Biochemistry, Memorial University of Newfoundland, St. John's, Newfoundland, Canada

**Keywords:** Glycine cleavage system (GCS), One-carbon metabolism, Glycine, Folate, Neural tube defects

## Abstract

The glycine cleavage system (GCS) is a complex of four enzymes enabling glycine to serve as a source of one-carbon units to the cell. We asked whether concentrations of glycine, dimethylglycine, formate, and serine in blood are influenced by variation within GCS genes in a sample of young, healthy individuals. Fifty-two variants tagging (r^2^ < 0.9) the four GCS genes were tested; one variant, *GLDC* rs2297442-G, was significantly associated (*p* = .0007) with decreased glycine concentrations in serum.

## 1 Introduction

The glycine cleavage system (GCS) is a crucial component of mitochondrial one-carbon metabolism (OCM). The GCS involves four distinct enzymes: glycine decarboxylase (*GLDC*), aminomethyltransferase (*AMT*), glycine cleavage system protein H (*GCSH*) and dihydrolipoamide dehydrogenase (*DLD*). These enzymes act sequentially in the mitochondrion to cleave glycine obtained from the cytoplasm or from the breakdown of serine in the mitochondrion to produce *N*,*N*-methylene tetrahydrofolate (CH_2_-THF), an active folate derivative that contributes to the transfer of one‑carbon units in cellular reactions [1].

Loss-of-function mutations in two GCS genes (*GLDC* and *AMT* [2]) cause non-ketotic hyperglycinemia (NKH), a rare recessive disease. Additionally, two NKH patients have been observed to have a complex rearrangement of *GCSH* [3]. Affected patients suffer from neurological impairments, seizures, and developmental delay, suggesting that the GCS is important for normal brain development and function [4]. In a study population of patients from the UK, Sweden and Japan, two of the genes encoding GCS enzymes, *AMT* and *GLDC,* were found to have missense variants associated with neural tube defects (NTDs) [5]. Similarly, variants in these genes were identified in a study of American patients with myelomeningocele, a type of NTD [6]. Finally, *GLDC* rs14742391 (p.Ser951Tyr) has been observed in a patient with NKH [2] and a patient with anencephaly, a type of NTD [7].

Although genome-wide association studies (GWASs) can identify gene variants that influence traits, they are often underpowered to detect signals [8] from real but small effects of common alleles, or large effects of rare alleles. GWASs have been carried out for serum glycine, identifiying a single nucleotide polymorphism (SNP) in carbamoyl-phosphate synthase 1 (rs715) [[9], [10], [11], [12]]. Blood serine has been associated with a SNP in phosphoglycerate dehydrogenase (rs477992) [9, 12, 13]). To test for other genetic modifiers of OCM that may have been missed by the GWAS method, we examined whether common variants in the genes of the GCS influence relevant metabolites in a healthy population.

## 2 Materials and methods

### 2.1 The Trinity Student Study (TSS) cohort

The TSS cohort comprises a population of 2232 healthy Irish students, as described [[14], [15], [16]]. Informed consent and ethical approval were obtained from all participants. Briefly, blood samples were collected into EDTA and clotting tubes, processed within 3 h, and stored at −80 °C before assaying. Glycine (interassay CV = 3.3%) and serine (interassay CV = 5.7%) in serum were measured with gas-chromatography tandem mass spectrometry (GC–MS/MS [17]) and plasma dimethylglycine (interassay CV <10%) by liquid-chromatography tandem mass spectrometry (LC-MS/MS [18]) by the laboratory of Bevital, Norway (www.bevital.no). Formate was measured using a newly developed GC–MS method [19]. A single control sample was frozen in aliquots for daily analysis with formate assays of participant serum samples. The day-to-day variation for this sample was 7.4% (*n* = 38). Genome-wide SNP genotyping was conducted at the Center for Inherited Disease Research (CIDR, USA) using Illumina 1 M HumanOmni1-Quad_v1-0_B chips.

### 2.2 Metabolite association analyses

Conservative (r^2^ < 0.9) tagging variants in the four GCS genes including 10 kb flanking regions were selected to test for association with levels of serum glycine, plasma dimethylglycine, serum serine, and serum formate. Log_10_ transformations of metabolite concentrations were used to meet normality assumptions. Linkage disequilibrium (LD) analyses were performed with Haploview; a total of 52 tag SNPs covering 93 variants were selected for association testing [20, 21]. For each SNP, association was tested using linear regression with a 1-df test (additive genetic model) or a 2-df test (genotypic model) (R statistical language and environment (version 3.3.2) [22], R package snpStats [23]). Bonferroni correction for 52 tests was applied to the nominal significance threshold (*p* < .05) to obtain a corrected significance threshold (*p* < .00096). Associations between log_10_ transformed metabolite data were assessed using Pearson's correlation coefficient (r).

### 2.3 GLDC expression association analyses

The public Gene-Tissue Expression resource (https://www.gtexportal.org/home) was used to search for associations between SNPs and *GLDC* mRNA levels (mRNA expression quantitative trait locus, eQTLs).

## 3 Results

The 52 tag SNPs in the four glycine cleavage genes (*GLDC*, *AMT*, *GCSH*, and *DLD*) were tested for association with blood levels of glycine, dimethylglycine, formate, and serine in 2232 young healthy Irish adults (Table 1). Serum glycine concentrations were significantly correlated with all the other metabolites measured. The strength of these associations varied from *r* = 0.52; *p* < .001 with serine to −0.07; *p* = .004 with formate. Only one GCS SNP is significantly associated with a metabolite. *GLDC* rs2297442 returns a *p*-value of 0.0007 following 1-df association testing with log_10_ transformed glycine concentration. The 2-df test of association is also significant (*p* = .0031), nominally.

**Table 1:** Metabolite characteristics in the TSS and by genotype group of its most associated GCS variant.

	Serine (μM)	Glycine (μM)	Dimethylglycine (μM)	Formate (μM)
Mean +/− SD	147.2 +/− 23.9	293.7 +/− 63.9	4.17 +/− 1.22	25.9 +/− 7.8
Median	145.6	288.1	3.99	24.8
Number⁎	2210	2210	2227	1535
Top SNP	rs2297442	rs2297442	rs16924717	rs7031325
p-value⁎⁎	0.0225	0.0007	0.0047	0.0133
				
Genotype (No.)	AA (1198)	AA (1198)	AA (2130)	TT (647)
Mean +/− SD	148.1 +/− 24.5	297.6 +/− 65.8	4.2 +/− 1.2	26.1 +/− 8.2
				
Genotype (No.)	AG (848)	AG (848)	AG (95)	TC (701)
Mean +/− SD	146.9 +/− 23.7	290.6 +/− 63.2	4.6 +/− 2	25.7 +/− 7.1
				
Genotype (No.)	GG (164)	GG (164)	GG (2)	CC (187)
Mean +/− SD	143.1 +/− 23.4	281.1 +/− 53.7	3.8 +/− 2.7	25.9 +/− 9

⁎ Number of participants with metabolite and genotype values.

⁎⁎ The Bonferroni-corrected threshold for significance is *p* < .00096.

We then sought to replicate the association between rs2297442-G and decreased levels of circulating glycine by examining results from two recent GWASs in which glycine was measured. Our observation was replicated in two studies of Finnish individuals (*n* = 16,506, ß = −0.039, *p* = .0014 [24]; *n* = 8545, ß = −0.039, *p* = .028 [25]).

**Fig. 1:**
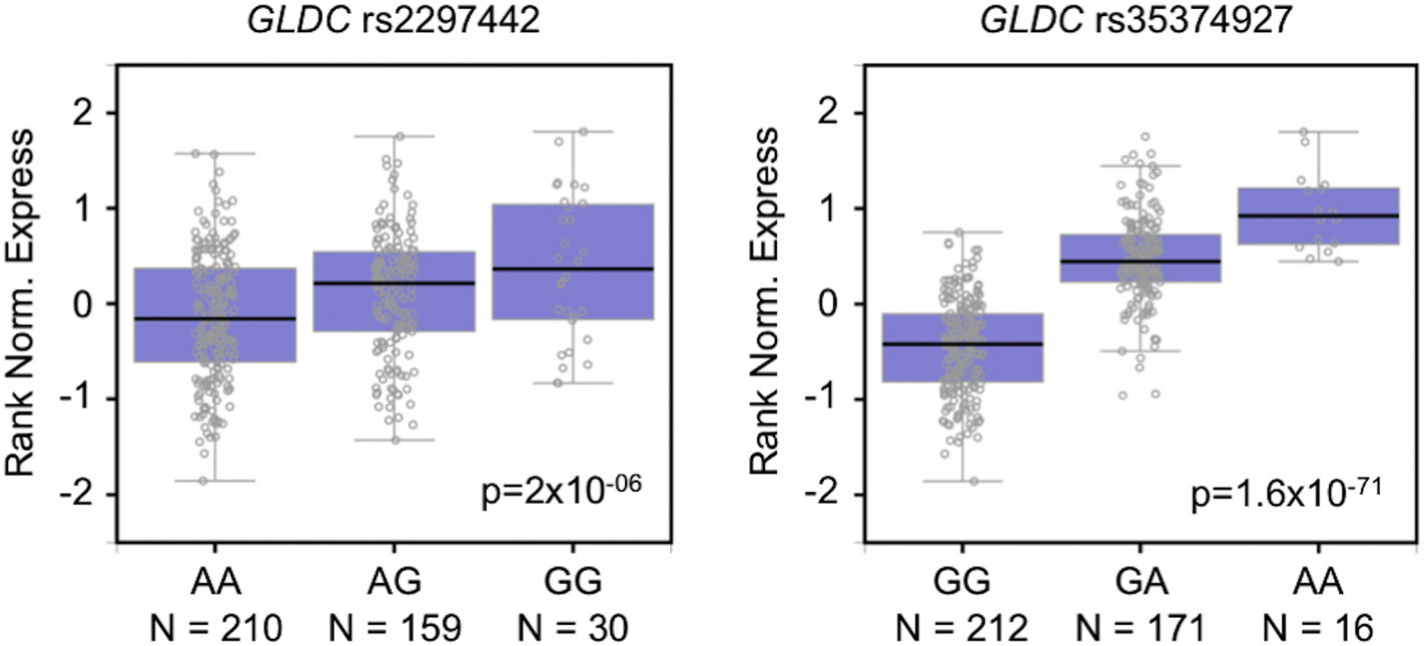
Association of rs2297442 (left) or rs35374927 (right) with *GLDC* mRNA levels in human thyroid. In these graphs generated by GTEx, participants are grouped by genotype and plotted for ranked normalized expression of *GLDC* mRNA in thyroid. Unadjusted *p*-values for linear regression analyses are shown. *GLDC* rs2297442 effect size (i.e., slope of the linear regression) = 0.3. *GLDC* rs35374927 effect size =1.0.

We also asked if *GLDC* mRNA expression levels are influenced by rs2297442 genotype. A Gene-Tissue Expression (GTEx) query of rs2297442 did not reveal a strong or consistent effect on *GLDC* mRNA levels across multiple tissues. In contrast, several other *GLDC* SNPs strongly influenced *GLDC* mRNA levels in several tissues (*p* < 1 × 10^−40^). For example, in thyroid tissue, *GLDC* rs35374927 is estimated to have a large impact on *GLDC* mRNA levels compared to *GLDC* rs2297442 (Fig. 1). These eQTLs of strong effect reside in LD blocks near the 5′ end of the gene, as opposed to the SNPs in the LD block at the 3′ end of the gene where rs2297442 is located.

## 4 Discussion

The results of these association tests confirm that variation in *GLDC* can influence serum glycine concentrations. The variant reported here, *GLDC* rs2297442, is a noncoding polymorphism found in the seventh of the ten introns in this gene*.* The GTEx data revealed that other, unlinked SNPs in the gene have a larger impact on *GLDC* mRNA levels, albeit in limited, specific tissues. We conclude that rs2297442 does not influence serum glycine levels via modulation of *GLDC* transcript levels. It is possible that rs2297442 is simply linked to the causal SNP. A search for a linked, exonic SNP in a population of European ancestry [26] failed to identify any strongly related variants (r^2^ < 0.2).

These results are consistent with the presence of a common SNP in *GLDC* that may increase the activity or alter the tissue distributions of this enzyme, causing reduced levels of circulating glycine. We predict that this effect on serum glycine concentrations may not be clinically relevant in this healthy population; however, it may contribute to diseases with a complex mode of inheritance, where combinations of many environmental and genetic factors of small individual effect must converge. The known link between folic acid supplementation and neural tube defect (NTD) prevention implicates perturbations of OCM as a key risk factor. In a study of 258 NTD patients, 27 single-base substitutions were discovered in *GLDC,* six of which influenced enzyme activity in an in vitro system [5]. The association between *GLDC* rs2297442 and serum glycine levels in the healthy populations in this study and others identifies a candidate locus for studies investigating the genetic basis of NKH, NTDs or any pathophysiology involving OCM.
